# P02-17 Promoting physical activity for mental health in the EU: Development and implementation of practice guidelines from the SPHERE EU project 2019-2020

**DOI:** 10.1093/eurpub/ckac095.036

**Published:** 2022-08-29

**Authors:** Diane Crone, Paul Sellars

**Affiliations:** Cardiff Metropolitan University, Cardiff, United Kingdom

**Keywords:** physical activity, mental health, recommendations, guidelines

## Abstract

**Issue/problem:**

Despite physical activity having an established evidence base for mental health problems there is mixed practice across the EU regarding its role in the support of mental health problems. The SPHERE EU project (Sport Healing Rehabilitation; 2019-2020 http://www.ecos-europe.com/sphere/erasmus-project/) was established to draw together academics, practitioners and psychiatrists, to develop practical guidelines and case study examples for their implementation.

**Description of the problem:**

To support the use of physical activity for mental health promotion, a critical review of evidence was conducted to inform the development of Practice Guidelines with case study examples of practice.

**Results (effects/changes):**

Practice Guidelines include 17 recommendations relating to physiological, psychological and social dimensions of mental health rehabilitation. The presentation will outline the practical, evidence-based guidelines designed for practitioners and evaluators which can be used to support the use of physical activity for mental health in the EU.

**Lessons:**

Practical, evidence-based guidelines for practice must be flexible to allow for individual differences and preferences, settings (clinical or community), and facility and equipment availability. This aim of these Guidelines are to enable professionals involved in health enhancing physical activity for mental health to have that evidence translated into practice, in a form that can be transferred to their setting and country.

**Main messages:**

Despite physical activity being widely understood to have an important role in the lives of people with mental health problems and other common co-morbidities for this population group, guidelines remain scarce. The SPHERE project has addressed this dearth and provides practical and pragmatic recommendations for physical activity for people with mental health problems in the future.

